# Supplementary material for: Magnetization switching in polycrystalline Mn3Sn thin film induced by self-generated spin-polarized current
Source: Nat Commun. 2022 Sep 30;13:5744. doi: 10.1038/s41467-022-33345-2 (PMC9525633; doi:10.1038/s41467-022-33345-2)
Supplement: Supplementary file 1 — Supplementary Information [file 41467_2022_33345_MOESM1_ESM.pdf]

Magnetization switching in polycrystalline Mn_3Sn thin film induced by self-generated spin-polarized current

Hang Xie[1], Xin Chen[1], Qi Zhang[1,2], Zhiqiang Mu[3], Xinhai Zhang[2], Binghai Yan[4+], and Yihong Wu[1*]

[1]Department of Electrical and Computer Engineering, National University of Singapore, Singapore 117583, Singapore

[2]Department of Electrical and Electronic Engineering, Southern University of Science and Technology, Xueyuan Rd. 1088, Shenzhen 518055, China

[3]State Key Laboratory of Functional Materials for Informatics, Shanghai Institute of Microsystem and Information Technology, Chinese Academy of Sciences, Shanghai 200050, China

[4]Department of Condensed Matter Physics, Weizmann Institute of Science, Rehovot 7610001, Israel

[+]Email: binghai.yan@weizmann.ac.il
*Email: elewuyh@nus.edu.sg

S1. XRD patterns of Mn_3Sn in different structures

S2. Fitting and decomposition of *M-H* curves

S3. AHE and current-induced switching of Mn_3Sn at different t_{Mn3Sn}

S4. AHE and current-induced switching curves of Mn_3Sn at different temperature

S5. MOKE images of Mn_3Sn at different injection current

S6. Current-induced switching curves of Mn_3Sn at different in-plane assistant field

S7. Temperature dependence of resistivity and AHE of Mn_3Sn with different seed layers

S8. Current-induced switching of Ti/Mn_3Sn and Ti/Mn_3Sn/Ti

S9. Current-induced switching at different in-plane assistant field angle

S10. Correlation of switching ratio with AHE remanence

S1. XRD patterns of Mn_3Sn in different structures

The XRD patterns for Ta(2)/MgO(t_{MgO})/Mn_3Sn(60) at t_{MgO} =0, 4 are shown in Supplementary Fig. 1a and 1b, respectively. As can be seen, the peak positions remain the same for samples with different t_{MgO}, which indicates that the insertion of MgO layer does not affect the crystalline structure of Mn_3Sn. The main peaks include $(10\bar{1}1)$, $(11\bar{2}0)$, $(20\bar{2}0)$, (0002), $(20\bar{2}1)$, and $(20\bar{2}2)$. The angles between the hexagonal plane and the substrate plane for $(10\bar{1}1)$, $(11\bar{2}0)$, $(20\bar{2}0)$, (0002), $(20\bar{2}1)$, and $(20\bar{2}2)$ oriented crystallites are 46.8^o, 90°, 90°, 0°, 64.8^o, and 46.8^o, respectively. These angles are important for understanding the *M-H* loops to be discussed in Supplementary Information S2. We further performed XRD measurements for Mn_3Sn with Ti seed layer and Mn_3Sn directly grown on Si/SiO_2 substrate. As shown in Supplementary Fig. 1c and 1d, both show same peaks as Ta(2)/MgO(t_{MgO})/Mn_3Sn(60) samples. This suggests that the crystalline structure for 60 nm Mn_3Sn is same with various seed layers, but the detailed ratio of these planes might differ in different structures, which cannot be simply determined from the XRD profiles and may require the assistance of other characterizations such as *M-H* measurement (Supplementary Information S2) and temperature-dependent electrical measurement (Supplementary Information S7).

We also performed XRD measurements for MgO/Mn_3Sn(12) and Ti/Mn_3Sn(12), where the Mn_3Sn thickness is same as the one used for most current-induced switching in this study. As can be seen from Supplementary Fig. 1e and 1f, MgO/Mn_3Sn(12) shows obvious peaks of $(11\bar{2}0)$, which is missing in Ti/Mn_3Sn(12), while the peak of $(20\bar{2}1)$ exists in both samples. In addition, there is also a very small peak of $(20\bar{2}0)$ in both samples and a small peak of $(10\bar{1}1)$ in Ti/Mn_3Sn(12). This indicates that there are more grains with kagome plane perpendicular to the film plane in MgO/Mn_3Sn(12), while the crystalline plane of the grains in Ti/Mn_3Sn(12) is mostly tilted towards the film plane.

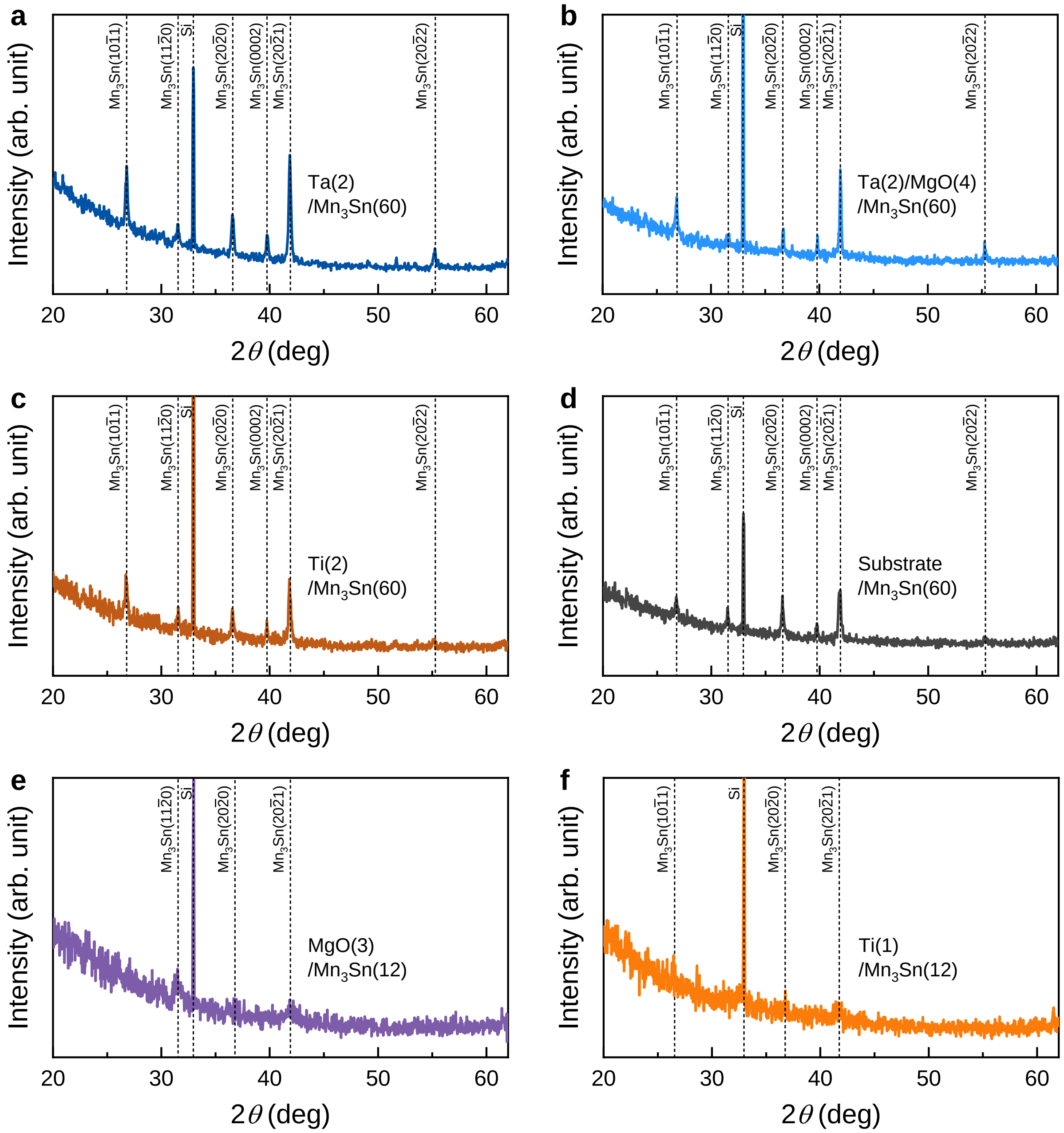

Supplementary Fig. 1 | XRD profiles of Mn_3Sn in different structures. a-f, XRD patterns of Ta(2)/Mn_3Sn(60), Ta(2)/MgO(4)/Mn_3Sn(60), Ti(2)/Mn_3Sn(60), Substrate/Mn_3Sn(60), MgO(3)/Mn_3Sn(12), Ti(1)/Mn_3Sn(12) thin films, respectively.

S2. Fitting and decomposition of *M-H* curves

We find that the *M-H* loops of polycrystalline Mn_3Sn can be decomposed into sub-loops using the relation

$$M = \frac{2M_s}{\pi} \sum_{i=1}^{n} \alpha_i \,\mathrm{atan}[\beta_i (H \pm H_{ci})] \quad (1)$$

Here, M_s is the total saturation magnetization, n is the number of a particular type of crystallites with a distinctive crystalline orientation, α_i is a proportional coefficient to denote the contribution from crystallites with different orientations with $\sum_{i=1}^{n} \alpha_i = 1$, β_i indicates how responsive each component is to an applied field, and H_{ci} is the coercivity. The $\pm$ sign indicates the backward and forward sweeping curves, respectively. We find that both the in-plane and out-of-plane curves for all the samples can be fitted very well by assuming $n = 2$. Supplementary Fig. 2 shows the fitted out-of-plane *M-H* curves, from which we can see that the measured *M-H* loop is the superposition of two sub-loops as expressed in Supplementary Equation (1). The fitting parameters are given in Supplementary Table 1, from which we can see that the two sub-loops have distinct coercivity. Since Mn_3Sn has a large out-of-plane (i.e., kagome plane) anisotropy, we may assume that sub-loop #1 with smallest coercivity comes from crystallites with their kagome planes perpendicular to the substrate including $(11\bar{2}0)$, $(20\bar{2}0)$ and the equivalent crystal planes, sub-loop #2 is due to the contributions from $(20\bar{2}1)$, $(10\bar{1}1)$, $(20\bar{2}2)$ planes and their equivalent planes. We shall point out that, when the sweeping field is in *z*-direction, the contributions from (0002) or other kogome planes which are nearly parallel to the substrate are small and they are mostly subtracted out together with the diamagnetic background. From Supplementary Table 1, we can see that a relatively thick MgO underlayer (e.g., t_{MgO} = 4 nm) promotes the formation of $(11\bar{2}0)$ and $(20\bar{2}0)$ planes, which have their kagome planes perpendicular to the substrate, in particular when t_{MgO} = 4 nm.

Supplementary Table 1 | Fitting parameters for Ta(2)/Mn_3Sn(72), Ta(2)/MgO(2)/Mn_3Sn(60), Ta(2)/MgO(4)/Mn_3Sn(60), and Ti(2)/Mn_3Sn(60), respectively.

Sample	M_s (emu/cm^3)	α_1	α_2	β_1	β_2	H_{c1} (T)	H_{c2} (T)
Ta(2)/Mn_3Sn(72)	7.70	0.21	0.79	25.29	4.33	0.0075	0.43
Ta(2)/MgO(2)/Mn_3Sn(60)	17.24	0.61	0.39	13.74	7.36	0.0081	0.53
Ta(2)/MgO(4)/Mn_3Sn(60)	19.60	0.83	0.17	12.00	2.20	0.040	0.60
Ti(2)/Mn_3Sn(60)	14.79	0.62	0.38	19.84	6.41	0.0052	0.49

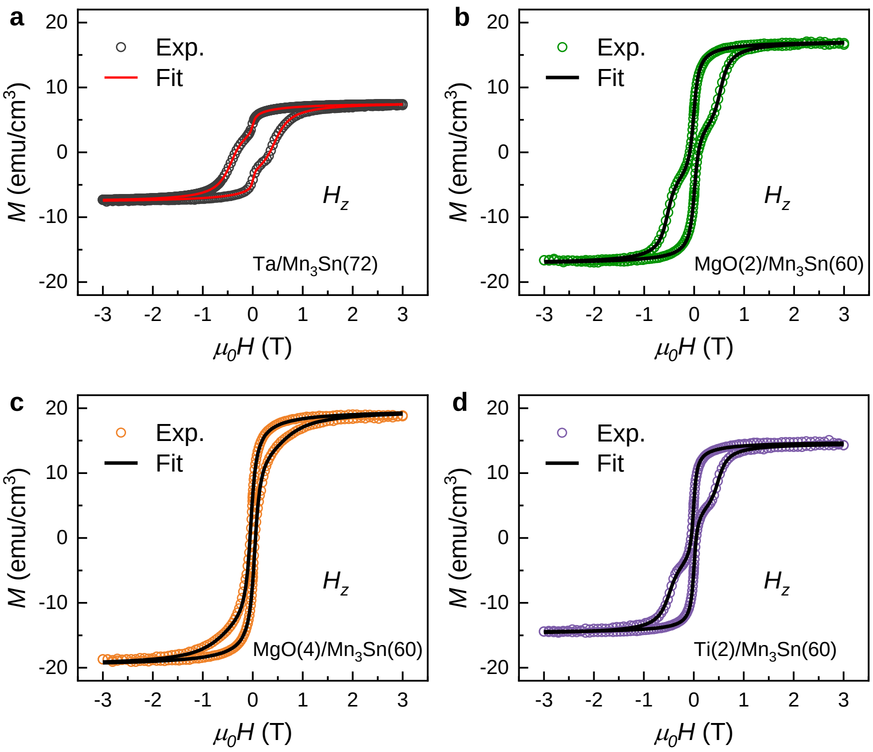

Supplementary Fig. 2 | Out-of-plane *M-H* curves of Mn_3Sn in different structures. a-d, Fitting of *M-H* curves for Ta(2)/Mn_3Sn(72), Ta(2)/MgO(2)/Mn_3Sn(60), Ta(2)/MgO(4)/Mn_3Sn(60), and Ti(2)/Mn_3Sn(60), respectively.

The magnetization of Mn_3Sn was also measured using an in-plane field with varying field angle $\varphi_{\mathrm{H}} = 0°$ (H_x), 45°, 90° (H_y), and 135°. Supplementary Fig. 3a-c show the in-plane *M-H* curves of Ta(2)/MgO(2)/Mn_3Sn(60), Ta(2)/MgO(4)/Mn_3Sn(60) and Ti(2)/Mn_3Sn(60), respectively. Here, $\varphi_{\mathrm{H}} = 0$ corresponds to one edge of the sample. We also fit these in-plane *M-H* curves using Supplementary Equation (1). As shown by the solid-lines in each figure, the in-plane *M-H* curves can also be fitted well using two independent magnetization components M_1 and M_2, which are plotted separately in Supplementary Fig. 3d. The fitting parameters are summarized in Supplementary Table 2. Unlike the *M-H* curve measured using a vertical field, the in-plane curve should contain the response from the (0002) plane and all other planes with a small angle with the applied field.

Although α_1/α_2 ratio varies slight between the two samples and also among the different directions, which might be caused by the finite out-of-plane field component due to sample misalignment, in general, we can see that there is no in-plane preferred orientation for the crystal grain. This would facilitate the IGSTT-based switching as illustrated in Fig. 5 of the main text.

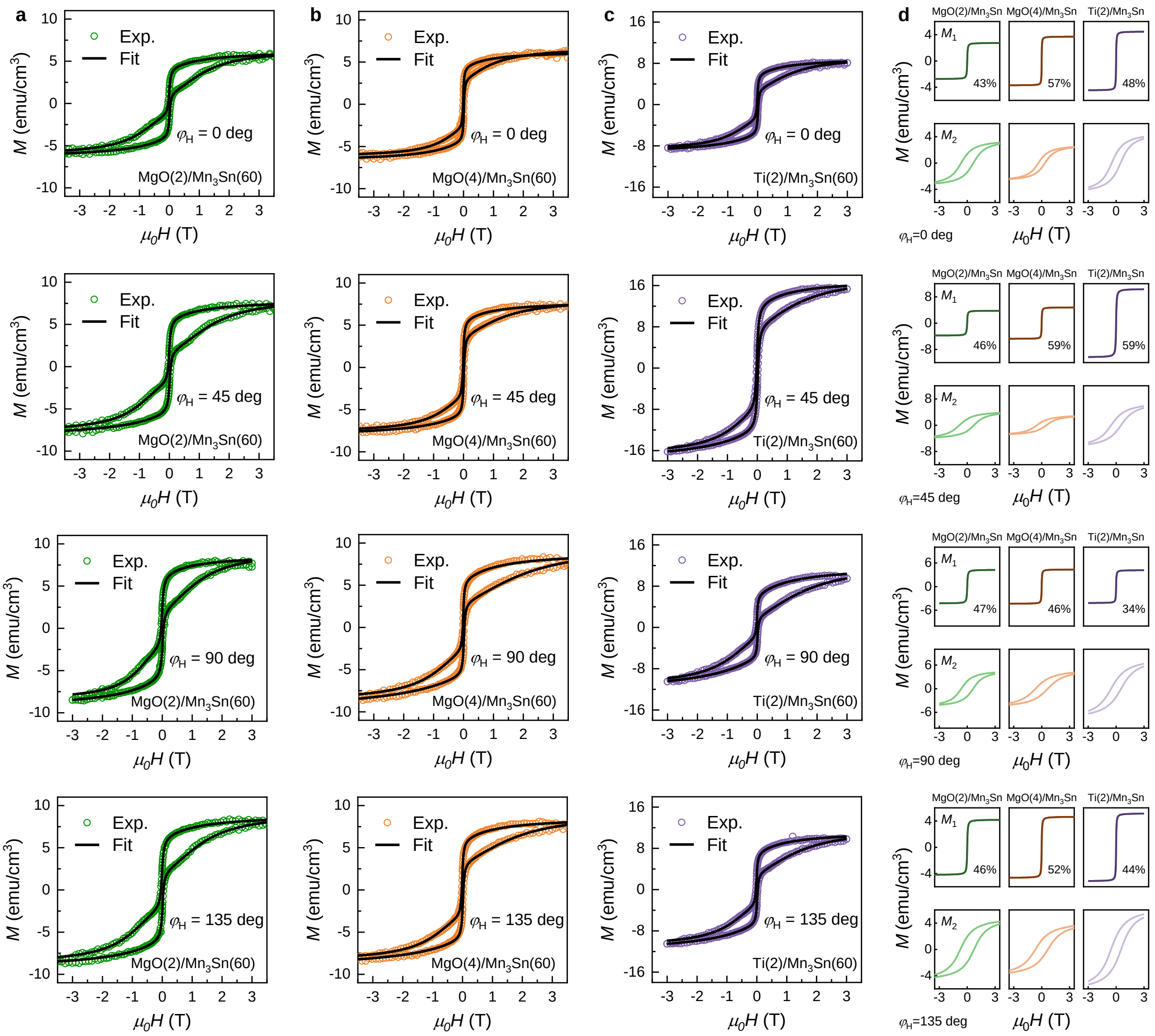

Supplementary Fig. 3 | In-plane *M-H* curves of Mn_3Sn in different structures. a-c, In-plane *M-H* curves measured at different field angle φ_H with their fitting for Ta(2)/MgO(2)/Mn_3Sn(60), Ta(2)/MgO(4)/Mn_3Sn(60) and Ti(2)/Mn_3Sn(60), respectively. **d,** Decomposed sub-loops of the *M-H* loops in **a-c.**

Supplementary Table 2 | Fitting parameters for Ta(2)/MgO(2)/Mn_3Sn(60), Ta(2)/MgO(4)/Mn_3Sn(60), and Ti(2)/Mn_3Sn(60) at different field angle φ_H, respectively.

Sample	φ_H (deg)	M_s (emu/cm^3)	α_1	α_2	β_1	β_2	H_{c1} (T)	H_{c2} (T)
Ta(2)/MgO(2)/Mn_3Sn(60)	0	6.41	0.43	0.57	26.97	1.03	0.0045	0.66
	45	8.22	0.46	0.54	23.72	0.96	0.0066	0.78
	90	9.13	0.47	0.53	22.60	1.11	0.0073	0.64
	135	9.22	0.46	0.54	24.05	0.94	0.0094	0.74

Ta(2)/MgO(4)/Mn_3Sn(60)	0	6.56	0.57	0.43	45.40	1.17	0.0099	0.36
	45	8.05	0.59	0.41	34.59	0.97	0.0099	0.51
	90	9.47	0.46	0.54	33.01	0.74	0.010	0.66
	135	8.99	0.52	0.48	29.94	0.82	0.011	0.67
Ti(2)/Mn_3Sn(60)	0	9.31	0.48	0.52	36.10	1.04	0.0094	0.47
	45	17.73	0.59	0.41	23.73	0.85	0.011	0.53
	90	12.28	0.34	0.66	34.20	0.79	0.0093	0.54
	135	11.78	0.44	0.56	33.17	0.91	0.011	0.54

S3. AHE and current-induced switching of Mn_3Sn at different t_{Mn3Sn}

Supplementary Fig. 4a shows the AHE curves of Ta(2)/MgO(2)/Mn_3Sn(t_{Mn3Sn}) with different t_{Mn3Sn} from 4 nm to 20 nm. As can be seen, the AHE in Mn_3Sn disappears at t_{Mn3Sn} = 4 nm. The current-induced switching measurement was also performed for these samples. Supplementary Fig. 4b and 4c show the current dependence of Hall resistance in an in-plane assistive field H_x of +600 Oe and -600 Oe, respectively. The switching amplitude overall increases with decreasing the Mn_3Sn thickness and evident switching is still observed at t_{Mn3Sn} = 6 nm. To estimate the critical current density, we firstly calculated the resistivity of Ta and Mn_3Sn from the longitudinal resistance R_{xx} of the devices. The R_{xx} at different t_{Mn3Sn} is shown in Supplementary Fig. 4d, which fits well with a reciprocal function of t_{Mn3Sn}. By fitting $R_{xx} - t_{Mn3Sn}$ using the expression $R_{xx} = (l/w)\left(t_{\mathrm{Ta}}/\rho_{\mathrm{Ta}} + t_{\mathrm{Mn_3Sn}}/\rho_{\mathrm{Mn_3Sn}}\right)^{-1}$, where l and w are the length and width of Hall bar device, t_{Ta} (fixed as 2 nm), ρ_{Ta}, $t_{\mathrm{Mn_3Sn}}$, $\rho_{\mathrm{Mn_3Sn}}$ are the thickness and resistivity of Ta and Mn_3Sn, we obtained ρ_{Ta} and $\rho_{\mathrm{Mn_3Sn}}$ as 680.6 and 225.9 μΩ cm, respectively. The Mn_3Sn resistivity is consistent with the reported value for either polycrystalline or single crystal Mn_3Sn thin film, while the relatively large Ta resistivity might be due to the small thickness. Supplementary Fig. 4e shows the critical current density in Ta layer (blue squares) and Mn_3Sn layer (red stars) based on the current distribution in each layer. The switching ratio is also extracted, which is shown in Supplementary Fig. 4f. We can find that the critical current density increases monotonically as the decrease of Mn_3Sn thickness, but the change is not very significant, which is around the level of 3 MA cm^{-2} in Ta layer. The larger critical current density at smaller t_{Mn3Sn} might be due to the increased switching ratio.

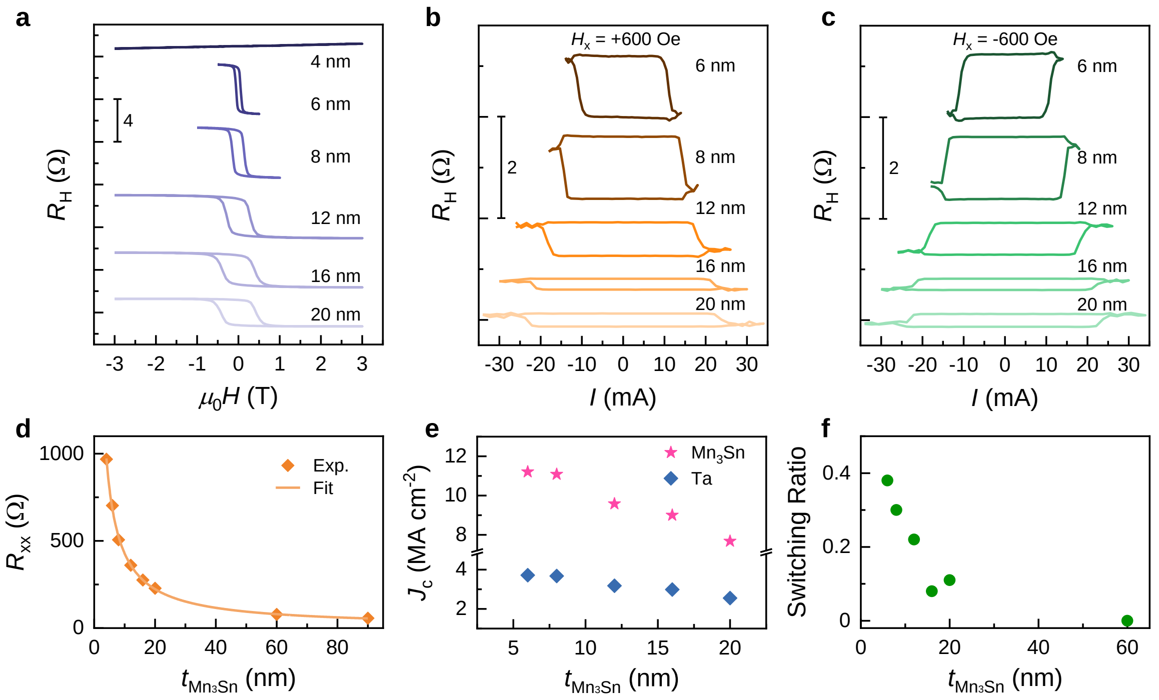

Supplementary Fig. 4 | AHE and current-induced switching in Ta/MgO/Mn_3Sn with varying Mn_3Sn thickness. **a**, Field dependence of Hall resistance of Ta(2)/MgO(2)/Mn_3Sn(t_{Mn3Sn}) with t_{Mn3sn} = 4 - 20 nm. **b-c,** Current dependence of Hall resistance of Ta(2)/MgO(2)/Mn_3Sn(t_{Mn3Sn}) with t_{Mn3Sn} = 6 - 20 nm under an in-plane assistive field H_x of +600 Oe and -600 Oe, respectively. **d,** Longitudinal resistance R_{xx} as a function of t_{Mn3Sn}. The solid line in **d** is the fitting result. **e-f,** Extracted critical current density J_c and switching ratio of Ta(2)/MgO(2)/Mn_3Sn(t_{Mn3Sn}) as a function of t_{Mn3Sn}.

As a control, AHE in Ta/Mn_3Sn was also studied. Supplementary Fig. 5a shows AHE curves of Ta/Mn_3Sn with varying Mn_3Sn thickness t_{Mn3Sn}. The extracted anomalous Hall resistivity ρ_H is plotted against t_{Mn3Sn} in Supplementary Fig. 5b, with the inset showing the Hall conductivity σ_H. The Hall resistance increases and the coercivity decreases with decreasing the thickness, which is similar to the results of Mn_3Sn with MgO underlayer. However, as can be seen, Ta/Mn_3Sn has a smaller Hall resistivity than Ta/MgO/Mn_3Sn at the same Mn_3Sn thickness. In addition, the AHE in Ta/Mn_3Sn becomes diminishingly small as t_{Mn3Sn} becomes less than 12 nm, while Ta/MgO/Mn_3Sn still shows large AHE at t_{Mn3Sn} = 6 nm. Therefore, the MgO underlayer helps to improve the magnetic properties of Mn_3Sn.

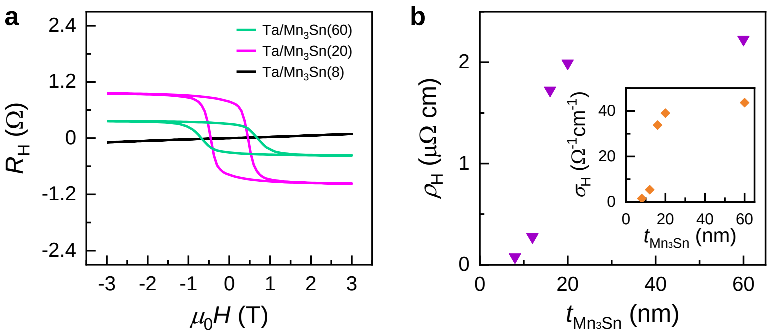

Supplementary Fig. 5 | AHE of Ta/Mn_3Sn with varying Mn_3Sn thickness. a, Field dependence of Hall resistance of Ta(2)/Mn_3Sn(t_{Mn3Sn}) at varied t_{Mn3sn} from 8 to 60 nm. **b,** Extracted ρ_H as a function of t_{Mn3Sn}. Inset: anomalous Hall conductivity as a function of t_{Mn3Sn}.

The switching polarity reversal in samples with thicker MgO layer is also observed at t_{Mn3Sn} = 8 nm. Supplementary Fig. 6a-b show the current-induced switching loops of Ta(2)/MgO(4)/Mn_3Sn(8) at H_x of +600 Oe and -600 Oe, respectively. As can be seen, the switching polarity is opposite to the switching polarity in samples with $t_{MgO} \leq 2$ nm.

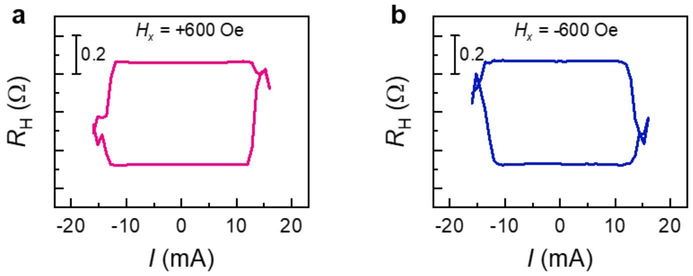

Supplementary Fig. 6 | Current-induced switching in Ta(2)/MgO(4)/Mn_3Sn(8). a-b, Current dependence of Hall resistance of Ta(2)/MgO(4)/Mn_3Sn(8) with an in-plane assistive field H_x of +600 Oe and -600 Oe, respectively.

S4. AHE and current-induced switching curves of Mn_3Sn at different temperature

Supplementary Fig. 7a shows the field dependence of Hall resistance for Ta(2)/MgO(2)/Mn_3Sn(12) at different temperature from 180 K to 400 K. Evident AHE with clear hysteresis maintains from 220 K to 400 K in Mn_3Sn. Both the amplitude and coercivity increase as the temperature decreases from 400 K to 240 K. As the temperature further decreases from 240 K, the AHE curve starts to become more slanted with decreased Hall resistance amplitude. At temperature lower than 200 K, the AHE almost disappears. The corresponding current-induced switching loops at different temperature with an in-plane

assistive field H_x of +400 Oe and -400 Oe are shown in Supplementary Fig. 7b and 7c, respectively. As can be seen, the switching amplitude follows a similar trend as the AHE amplitude, which keeps increasing as the temperature decreases from 400 K to 240 K below which it quickly decreases to nearly zero from 240 K to 200 K.

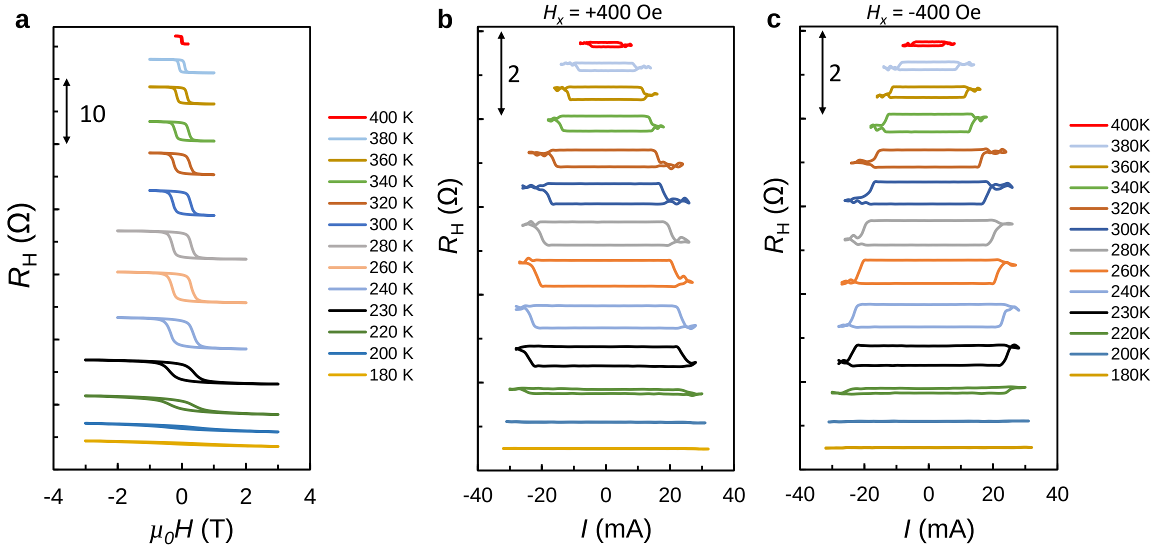

Supplementary Fig. 7 | AHE and current-induced switching in Ta/MgO/Mn_3Sn at different temperature. a, Field dependence of Hall resistance of Ta(2)/MgO(2)/Mn_3Sn(12) at varying temperature from 180 K to 400 K. **b-c,** Current dependence of Hall resistance of Ta(2)/MgO(2)/Mn_3Sn(12) at varying temperature from 50 K to 400 K with an in-plane assistive field H_x of +400 Oe and -400 Oe, respectively.

S5. MOKE images of Mn_3Sn at different injection current

Supplementary Fig. 8a and 8b show the current-induced switching process in Mn_3Sn as the current is swept from 0 mA to 26 mA at a H_x of +460 Oe and -460 Oe, respectively. The current pulse width is fixed at 5 ms. A negative current of -26 mA was first applied to initiate the magnetic state in Mn_3Sn. From the MOKE imaging, the switching starts to occur around +17.5 mA, which is consistent with electrical measurement result shown in Fig. 2b-c of the main text. As the current further increases from +17.5 mA, the colour of the Hall bar becomes darker at H_x= +460 Oe (Supplementary Fig. 8a) and brighter at H_x= -460 Oe (Supplementary Fig. 8b). In the whole switching process till it saturates at +26 mA, the change in colour is gradual and homogeneous without clear domain wall propagation observed. Therefore, the switching in polycrystalline Mn_3Sn occurs in a gradual rotation manner at individual crystalline grains.

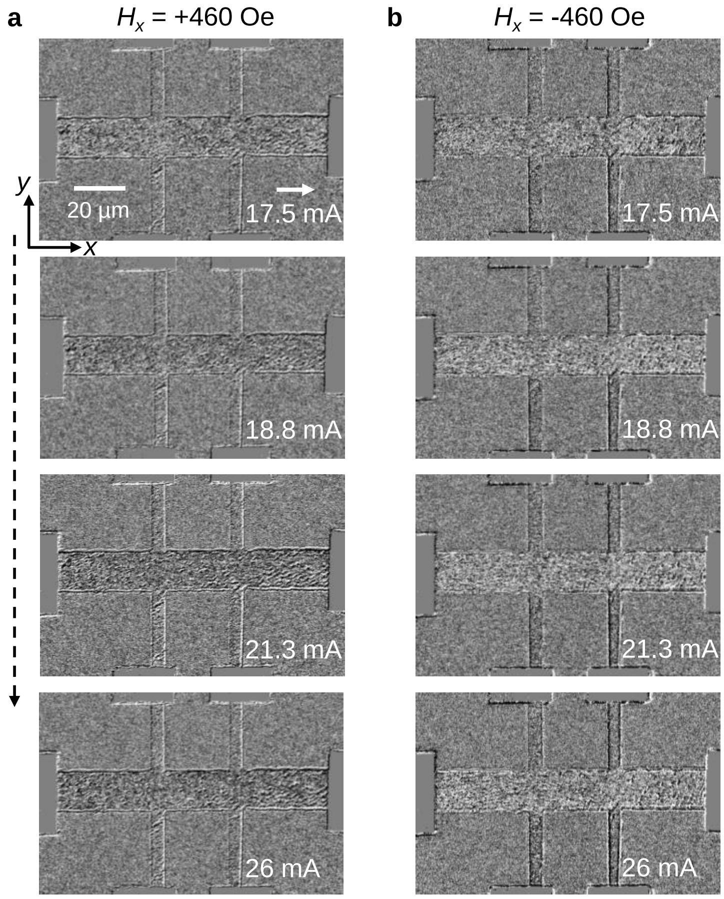

Supplementary Fig. 8 | MOKE images of current-induced switching process in Mn_3Sn. a-b, The switching process captured by MOKE at an in-plane assistive field H_x of +460 Oe and -460 Oe, respectively. The dashed arrow indicates the time sequence of the images.

S6. Current-induced switching curves of Mn_3Sn at different in-plane assistant field

Supplementary Fig. 9a shows the current-induced switching curves of Ta(2)/MgO(3)/Mn_3Sn(12) at varied H_x from -1000 Oe to 1000 Oe. The extracted switching ratio from switching loops as a function of H_x is displayed in Supplementary Fig. 9b. We can find that the switching presents nearly zero switching ratio at $H_x = 0$ Oe, which gradually increases as the increase of H_x and then peaks at $H_x = 600$ Oe. Afterwards, it slowly decreases as the further increase of H_x. The field dependence of the current-induced switching is overall consistent with the reported result in HM/Mn_3Sn bilayer structure[1-3]. There is no critical assistant field for switching. For conventional HM/FM bilayers with PMA, there is a need to use an in-plane assistant field to break the symmetry. However, in the present case, we believe the main role of the assistant field is to change the spin polarization direction of the polarizer rather than break the symmetry of the analyzer.

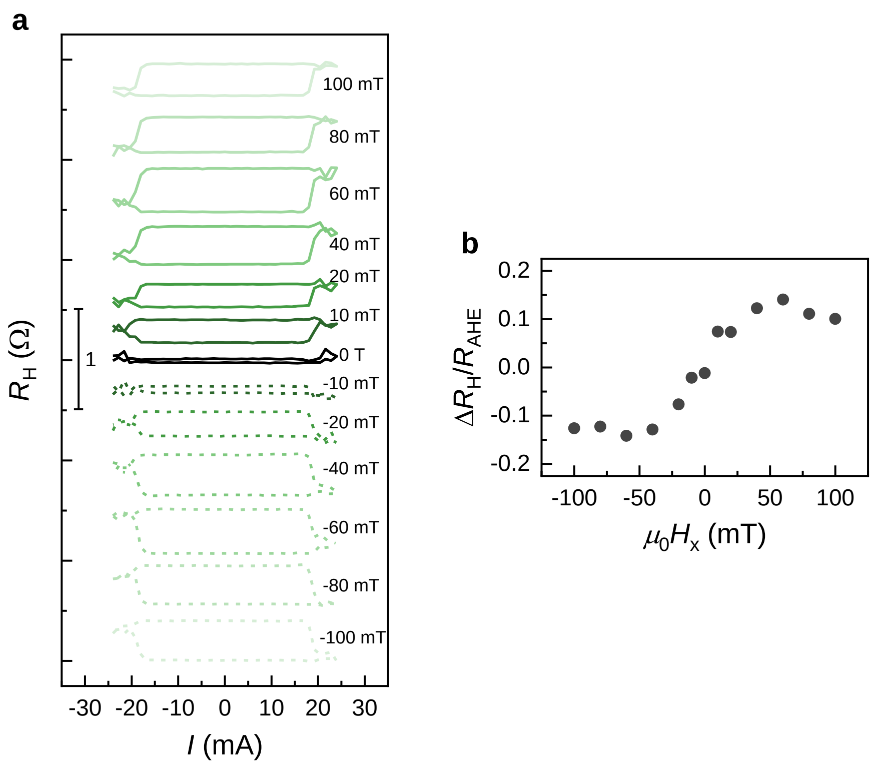

Supplementary Fig. 9 | Current-induced switching of Mn_3Sn at varying in-plane assistant field. a, Field dependence of Hall resistance of Ta(2)/MgO(3)/Mn_3Sn(12) at varying in-plane assistant field from -100 mT to 100 mT. **b,** Extracted switching ratio from the switching loops in **a** as a function of H_x.

S7. Temperature dependence of resistivity and AHE of Mn_3Sn with different seed layers

To gain some insight of the seed layer effect on its crystalline orientations, we also examined the temperature dependence of longitudinal resistivity and Hall conductivity of 12 nm Mn_3Sn with different seed layers (MgO and Ti underlayers with different thickness t_{Ti} and t_{MgO}). Supplementary Fig. 10a and 10b show the relative change of longitudinal resistivity $\rho_{xx}/\rho_{xx,300K}$ with respect to the value at 300 K and Hall conductivity $\sigma_H(H = 0)$ at zero field (corresponding to AHE contributed by non-collinear AFM phase) as a function of temperature, respectively. As can be seen, overall, except Ti(5)/Mn_3Sn, all the samples show decreased ρ_{xx} at lower temperature. As for Hall conductivity, phase transition of non-collinear AFM is observed for all the structures. However, Mn_3Sn grown on Ti layer shows higher transition temperature of around 260 K compared to MgO/Mn_3Sn with transition temperature around 220 K. According to temperature dependence of σ_H for single crystal (field applied along kagome plane) and polycrystalline Mn_3Sn, single crystal has much lower transition temperature at around 50 K compared to the latter[4,5]. This implies that the lower transition temperature in

MgO/Mn_3Sn might be due to the existence of more group i) grains, whose kagome planes are parallel to z-axis (the direction of applied field in AHE measurement). This corroborates well with the XRD and M-H measurement results shown in Supplementary Information S1 and S2. From the temperature dependent AHE, we also find that MgO/Mn_3Sn still shows negative AHE at low temperature (50 K to 108 K) as shown in Supplementary Fig. 10d, while Ti/Mn_3Sn shows ferromagnetic type of AHE (Supplementary Fig. 10e-f), suggesting the Mn_3Sn transition into glassy FM phase[6]. As the increase of Ti thickness, the amplitude of FM-phase AHE at low temperature further increases. In addition, as shown in Supplementary Fig. 10g-i, Ti/Mn_3Sn also shows evident magnetoresistance (MR) at 50 K, which is however absent in MgO/Mn_3Sn, further indicating the FM phase in Ti/Mn_3Sn at low temperature. This magnetic property of Ti/Mn_3Sn corresponds well with that of epitaxial Mn_3Sn (0001) film[6], though Ti/Mn_3Sn still shows negative AHE at room temperature while Mn_3Sn (0001) film only shows ordinary Hall effect at room temperature. This implies that the Ti seed layer, especially a thick Ti layer, facilitates the formation of (0001) crystalline plane (with c-axis along z-direction) in Mn_3Sn. For Ti/Mn_3Sn with thinner Ti layer (t_{Ti} = 1, 2 nm), the relatively large AHE at room temperature can be explained by the fact that there are still many grains with the crystalline planes of $(10\bar{1}1)$, $(20\bar{2}2)$ and $(20\bar{2}1)$ (group ii grains), whose c-axis is tilted towards but not completely aligned to z-direction. This again suggests that the different seed layers lead to different crystalline orientations in Mn_3Sn, e.g., Mn_3Sn with MgO seed layer has more group i) grains with $(11\bar{2}0)$ and $(20\bar{2}0)$ orientations, and Mn_3Sn with Ti seed layer has more group ii) and group iii) grains with $(10\bar{1}1)$, $(20\bar{2}2)$, $(20\bar{2}1)$, and (0002) orientations.

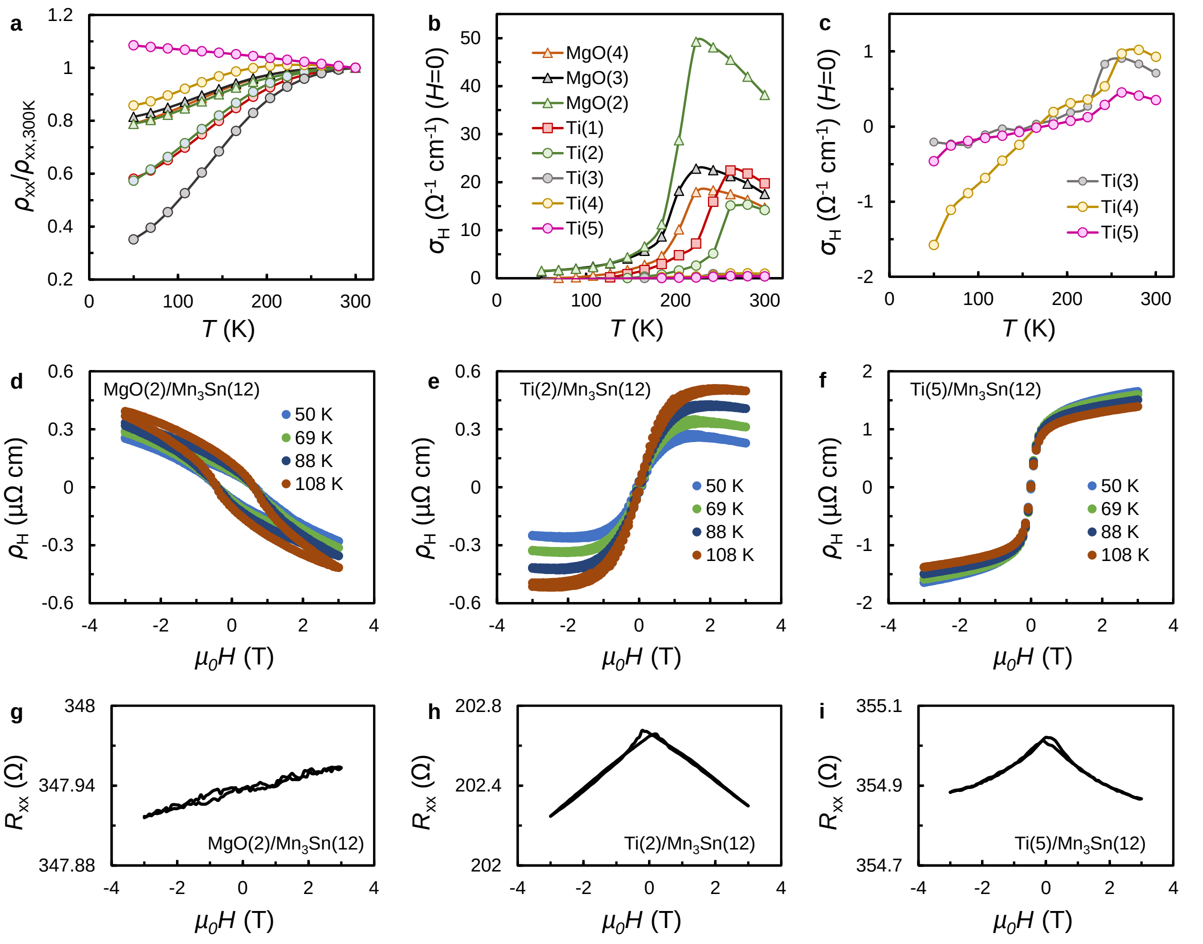

Supplementary Fig. 10 | Temperature dependence of resistivity and AHE of Mn3Sn with varying seed layers. a, Relative change of longitudinal resistivity $\rho_{xx}/\rho_{xx,300K}$ with respect to the value at 300 K as a function of temperature. **b-c,** Hall conductivity at zero field σ_{H} $(H = 0)$ of Mn_3Sn with different seed layers as a function of temperature. **d-f,** Hall resistivity as a function of magnetic field for MgO(2)/Mn_3Sn(12), Ti(2)/Mn_3Sn(12), and Ti(5)/Mn_3Sn(12), respectively. **g-i,** Longitudinal resistance as a function of field at 50 K for MgO(2)/Mn_3Sn(12), Ti(2)/Mn_3Sn(12), and Ti(5)/Mn_3Sn(12), respectively.

S8. Current-induced switching of Ti/Mn_3Sn and Ti/Mn_3Sn/Ti

As mentioned in the main text, it is unlikely that there is significant spin-current generation or spin accumulation at Ti/Mn_3Sn interface. Nevertheless, to exclude the interface effect at Ti/Mn_3Sn, we performed measurement in samples of Ti(t_{Ti})/Mn_3Sn(12) with different Ti thickness t_{Ti} from 1 nm to 5 nm, and Ti(1)/Mn_3Sn(12)/Ti(1) with symmetric structure. Supplementary Fig. 11a shows the AHE resistivity of Ti(t_{Ti})/Mn_3Sn(12), from which the AHE of Mn_3Sn keeps decreasing as the increase of Ti thickness. When t_{Ti} is larger than 2 nm, the

AHE becomes negligibly small and almost submerged by ordinary Hall effect signal (as shown in Supplementary Fig. 11b), which might be because the Ti layer facilitates the formation of (0001) crystalline plane (with *c*-axis along *z*-direction) in Mn_3Sn, especially a thick Ti layer, as has been discussed in Supplementary S7. Therefore, we could only perform current-induced switching for Ti(1)/Mn_3Sn(12). From Supplementary Fig. 11d-e, evident switching is also shown in Ti(1)/Mn_3Sn(12), with comparable switching ratio (~18%) and switching current (~20 mA) to the Ti(2)/Mn_3Sn(12) case. Therefore, decreasing the Ti thickness does not lead to significant increase of switching ratio or decrease of switching current. Furthermore, for Ti(1)/Mn_3Sn(12)/Ti(1) sample with more symmetric structure than Ti/Mn_3Sn, the switching (as displayed in Supplementary Fig. 11g-h) also shows same polarity, similar level of switching ratio (~21%) and switching current (~20 mA) as the Ti(1,2)/Mn_3Sn(12) samples, despite the existence of both upper and lower Ti/Mn_3Sn interfaces. If the switching is induced by spin current from the interface effect, we would expect nearly zero switching, or at least much smaller switching ratio in Ti(1)/Mn_3Sn(12)/Ti(1). However, the experiment result is very contrary to it. Combined with the switching results in Mn_3Sn/MgO and MgO/Mn_3Sn/MgO, it is suggested that the switching polarity is not related to the interface, and instead, it is the type of seed layer under Mn_3Sn that plays an important role in determining the switching polarity in Mn_3Sn.

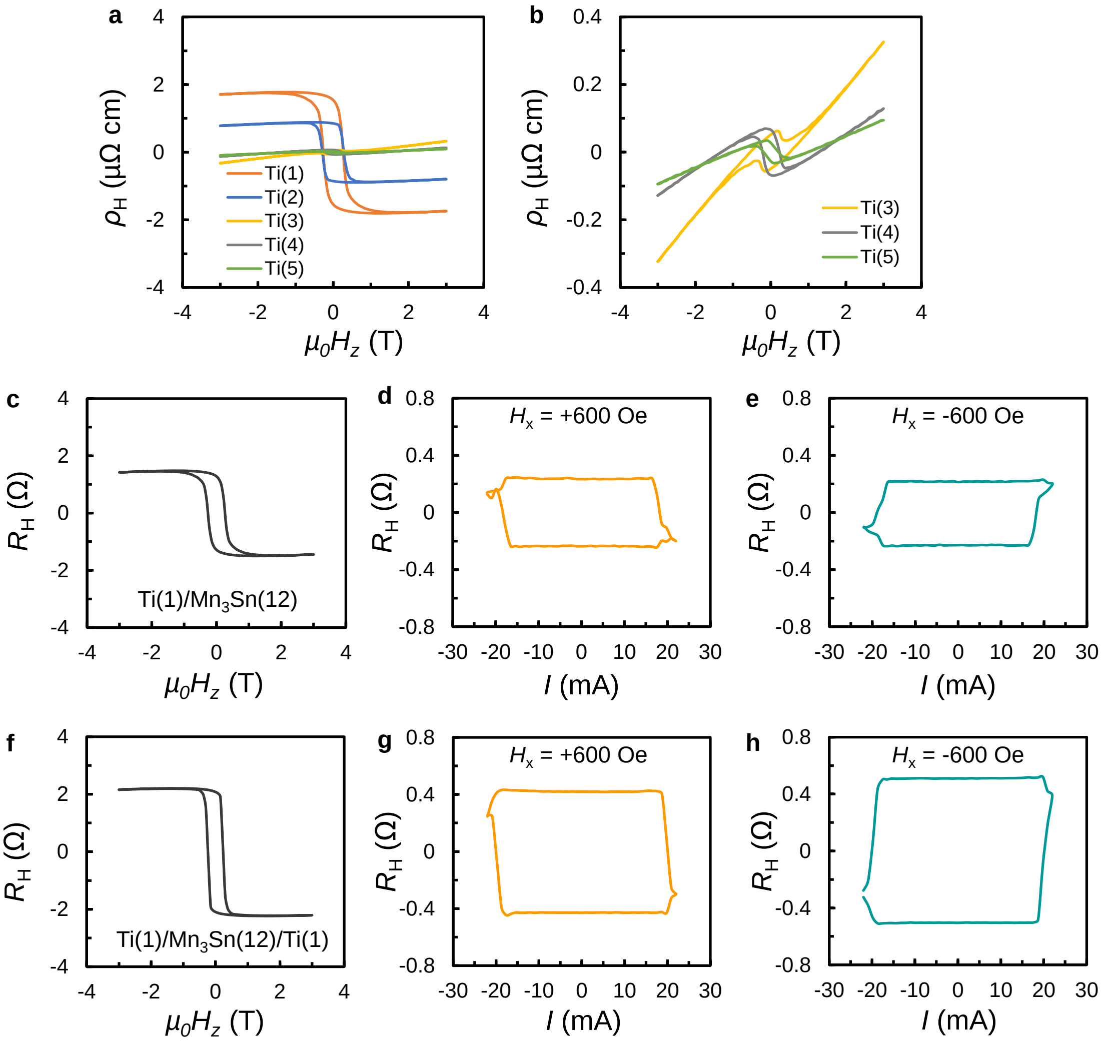

Supplementary Fig. 11 | AHE and Current-induced switching in Ti/Mn_3Sn and Ti/Mn_3Sn/Ti. a-b, Field dependence of Hall resistivities of Ti(t_{Ti})/Mn_3Sn(12) with Ti thickness t_{Ti} ranging from 1 nm to 5 nm. **b** is a zoom-in plot of $\rho_H - H_z$ for Ti(3,4,5)/Mn_3Sn(12). **c, f,** Field dependence of Hall resistance of Ti(1)/Mn_3Sn(12) and Ti(1)/Mn_3Sn(12)/Ti(1), respectively. **d-e,** Current dependence of Hall resistance of Ti(1)/Mn_3Sn(12) with an in-plane assistant field H_x of +600 Oe and -600 Oe, respectively. **g-h,** Current dependence of Hall resistance of Ti(1)/Mn_3Sn(12)/Ti(1) with an in-plane assistant field H_x of +600 Oe and -600 Oe, respectively.

S9. Current-induced switching at different in-plane assistant field angle

We also performed current-induced switching under an in-plane field with varying field-angle φ_H and a fixed amplitude of 400 Oe ($\varphi_H = 0$ corresponds to H_x). Supplementary Fig. 12a shows the change in Hall resistance ΔR_H in current-induced switching as a function of φ_H for Ta(2)/MgO(2)/Mn_3Sn(8). As can be seen, the maximum switching amplitude occurs at 0° (+x)

and 180° (-x), which resembles the SOT-induced switching case. However, differently, we also observed switching at 90°(+y) and 270°(-y) as shown in Supplementary Fig. 12b, though the switching amplitude is only one tenth of the value at $\varphi_H = 0$. These results further support IGSTT-based magnetization switching in polycrystalline Mn_3Sn.

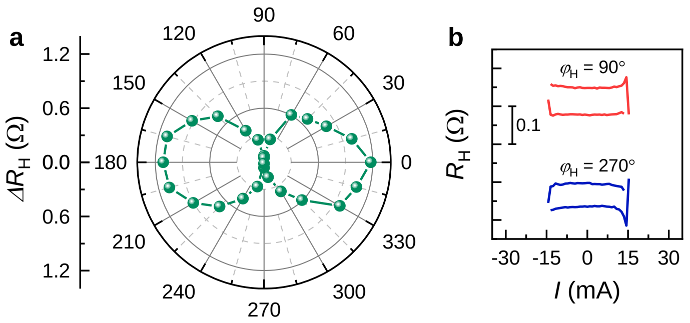

Supplementary Fig. 12 | Current-induced switching at varying in-plane assistant field angle. a, Field angle dependence of the Hall resistance change of Ta(2)/MgO(2)/Mn_3Sn(8) at 400 Oe. **b,** Hall resistance as a function of applied current amplitude of Ta(2)/MgO(2)/Mn_3Sn(8) in an external field at an angle of 90° (+y) and 270° (-y), respectively.

S10. Correlation of switching ratio with AHE remanence

As switching only occurs when the spin polarization is within the kagome plane, it is not possible to achieve 100% switching in polycrystalline samples as revealed by previous studies including the recent study using the nitrogen-vacancy sensor[2]. For HM/Mn_3Sn bilayer, where the spin current is mainly from heavy metal layer and the spin polarization direction is fixed, the more kagome planes that are parallel to the spin polarization the larger the switching ratio will be. For simplicity, we assume that the sample consists of group i) grains with randomly distributed orientations. In this case, we may estimate the switching ratio of HM/Mn_3Sn bilayers by assuming 1) the switching is based on conventional damping-like SOT, 2) only grains with crystalline plane coplanar with the spin polarization can be switched, and 3) grains which meet the polarization requirement are completely switched at sufficiently large current. Therefore, the estimated maximum switching ratio turned out to be $\frac{2}{\pi}\int_0^{\pi/2} \cos\varphi \, d\varphi = 0.64$. Here, φ is the misalignment angle of c-axis of kagome plane and spin polarization from HM metal layer. In fact, the maximum switching ratio for HM/Mn_3Sn bilayer structure is around 50% in reported study[7] and 61% in the our present work, which is close to the estimated value. The smaller switching ratio in previous studies might be due to the larger Mn_3Sn thickness used (30-40 nm).

On the other hand, when the switching is mainly induced by the self-generated spin current in Mn_3Sn, the switching ratio can be much smaller because a pair of neighbouring grains functioning as polarizer and analyzer with specific crystalline orientation is required. From current-induced switching in different structures, we find that the switching ratio overall presents a positive correlation with the AHE remanence $R_{\mathrm{H}(H=0)}/R_{\mathrm{AHE}}$ as shown in Supplementary Fig. 13, which is an indicator of degree of texturing of the non-collinear AFM film. The largest switching ratio achieved is around 20% in Ti/Mn_3Sn, where the AHE remanence is also largest. Nevertheless, we believe 100% switching is possible for small samples with a single grain in Ta/Mn_3Sn bilayer (via spin Hall current) or 2 grains in Mn_3Sn (via inter-gain spin torque transfer).

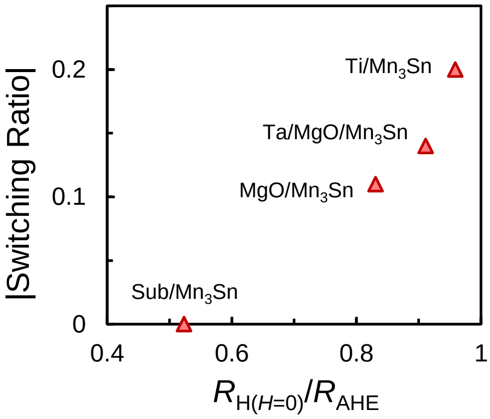

Supplementary FIG. 13 | Correlation of switching ratio with AHE remanence. The remanence $R_{\mathrm{H}(H=0)}/R_{\mathrm{AHE}}$ dependence of the switching ratio for Mn_3Sn with different underlayers. The thickness of Mn_3Sn fixed as 12 nm for all the structures.

References

1. Tsai, H. et al. Electrical manipulation of a topological antiferromagnetic state. *Nature* **580**, 608-613 (2020).

2. Yan, G. Q. et al. Quantum sensing and imaging of spin-orbit-torque-driven spin dynamics in noncollinear antiferromagnet Mn3Sn. *Adv. Mater.*, 2200327 (2022).

3. Krishnaswamy, G. K. et al. Time-dependent multistate switching of topological antiferromagnetic order in Mn3Sn. arXiv:2205.05309 (2022).

4. Nakatsuji, S., Kiyohara, N. & Higo, T. Large anomalous Hall effect in a non-collinear antiferromagnet at room temperature. *Nature* **527**, 212-215 (2015).

5. Higo, T. et al. Anomalous Hall effect in thin films of the Weyl antiferromagnet Mn3Sn. *Appl. Phys. Lett.* **113**, 202402 (2018).

6. Taylor, J. M. et al. Anomalous and topological Hall effects in epitaxial thin films of the noncollinear antiferromagnet Mn3Sn. *Phys. Rev. B* **101**, 094404 (2020).

7. Tsai, H. et al. Large Hall Signal due to Electrical Switching of an Antiferromagnetic Weyl Semimetal State. *Small Sci.* **1**, 2000025 (2021).
